# Spatio-Temporal Evolution, Prediction and Optimization of LUCC Based on CA-Markov and InVEST Models: A Case Study of Mentougou District, Beijing

**DOI:** 10.3390/ijerph19042432

**Published:** 2022-02-19

**Authors:** Yang Yi, Chen Zhang, Jinqi Zhu, Yugang Zhang, Hao Sun, Hongzhang Kang

**Affiliations:** 1 Key Laboratory of National Forestry and Grassland Administration on Ecological Landscaping of Challenging Urban Sites, National Innovation Alliance of National Forestry and Grassland Administration on Afforestation and Landscaping of Challenging Urban Sites, Shanghai Engineering Research Center of Landscaping on Challenging Urban Sites, Shanghai Academy of Landscape Architecture Science and Planning, Shanghai 200232, China; yy@shsyky.com; 2 School of Design, Shanghai Jiao Tong University, Shanghai 200240, China; 3 Shanghai Foundation Ding Environmental Technology Company, Shanghai 200063, China; joezhangchen@sina.com (C.Z.); sunhao930309@163.com (H.S.); 4 Jiangxi Institute of Ecological Civilization, School of Resources, Environmental & Chemical Engineering, Nanchang University, Nanchang 330031, China; zhujq@ncu.edu.cn; 5 Taihu Basin Monitoring Central Station for Soil and Water Conservation, Taihu Basin Authority of Ministry of Water Resources, Shanghai 200434, China; zhangyugang@tba.gov.cn

**Keywords:** LUCC, CA-Markov model, InVEST model, ecosystem service, Mentougou District

## Abstract

With the rapid advancement of urbanization and industrialization, the contradiction between the social economy and resources and the environment has become increasingly prominent. On the basis of limited land resources, the way to promote multi-objective comprehensive development such as economic, social development and ecological and environmental protection through structure and layout regulation, so as to maximize regional comprehensive benefits, is an important task of current land spatial planning. Our aim is to obtain land-use-change data in the study area using remote-sensing data inversion and multiple-model simulation. Based on land suitability evaluation, we predict and optimize the land use structure of the study area in 2030 and evaluate and compare ecosystem services. Based on remote-sensing images and eco-environmental data from 1985 to 2014 in the study area, land use/land cover change (LUCC) and future simulation data were obtained by using supervised classification, landscape metrics and the CA-Markov model. The ecosystem services were evaluated by the InVEST model. The analytic hierarchy process (AHP) method was used to evaluate the land suitability for LUCC. Finally, the LUCC in 2030 under two different scenarios, Scenario_1 (prediction) and Scenario_2 (optimization), were evaluated, and the ecosystem service functions were compared. In the last 30 years, the landscape in the study area has gradually fragmented, and the built-up land has expanded rapidly, increased by one-third, mainly at the cost of cropland, orchards and wasteland. According to the suitability evaluation, giving priority to the land use types with higher environmental requirements will ensure the study area has a higher ecosystem service value. The rapid development of urbanization has a far-reaching impact on regional LUCC. Intensive land resources need reasonable and scientific land use planning, and land use planning should be based on the suitability evaluation of land resources, which can improve the regional ecosystem service function.

## 1. Introduction

Under the influence of natural and human factors, land use/land cover change (LUCC) has had a latent influence on the material cycle and energy conversion in the ecological chain with the continuous advancement of urbanization. These impacts alter habitat quality and ecological change processes, and ultimately affect the ecosystem structure and function [1,2,3,4,5]. With the rapid economic development in recent years, the process of global urbanization has been accelerated, and the world’s urban population has reached half of the total population by 2020 [6]. The Chinese urbanization rate has increased from 36.22% in 2000 to 63.89% in 2020 [7]. By 2030, The Chinese urban population will account for about three-quarters of the total population [8]. Rapid urbanization has brought about a series of problems related to urban expansion, which might lead to inefficient LUCC, the heat island effect, soil erosion and human settlement deterioration [9,10,11]. According to the Millennium Ecosystem Assessment (MA), more than 60% of global Ecosystem Services (ES) were degraded or overutilized, and LUCC change caused by human activities was one of the important driving factors leading to the degradation of ES [12]. Therefore, monitoring, evaluating, forecasting and optimizing the evolution of LUCC and the resulting evolution of habitat and ES, so as to improve the quality of the urban ecological environment and the human settlement environment, was of great practical significance to maintain regional sustainable development [13,14].

In recent years, spatial-temporal forecasting has been used to help optimize the development direction of urban planning, so as to obtain the urban development dynamics and ES capacity in advance and provide timely feedback and adjustment, which has achieved good results [15,16]. Cellular Automata (CA), as one of the most important and widely used methods across many models, was the basis of many models, such as the Logistic-CA model [17], ANN-CA model [18], CLUE-S model [19] and FLUS model [20]. The CA-Markov model was a relatively successful simulation method, which combined the ability of the CA model to simulate the spatial changes of complex systems and the advantage of the Markov model in long-term prediction [21,22,23]. It not only improved the prediction accuracy of LUCC transformation, but also effectively simulated the spatial change of LUCC, which had good scientific and practical characteristics [24]. Jenerette et al. [25] used the CA-Markov model to study the impact of urbanization and population growth on landscape changes in Phoenix, Arizona (USA). Nourgolipour et al. [26] simulated the spatial pattern distribution of local palm plantations in the Kuala Lengyue region of Malaysia by combining CA-Markov and MCE models. Alcamo et al. [27] set four different scenarios and found that the tradeoff between different ES will be increasingly intensified in the 21st century. These studies not only provided a good research paradigm for LUCC management and planning at different scales, but also provide important information about the implications for the ecological processes and ES changed by LUCC.

The concept of ES was originally derived from the concept of the environmental service function proposed in the research on key environmental issues, and then gradually developed by Ehrlich et al. (1987) [28]. Since then, the value evaluation of global ES and natural capital published by Constanza et al. (1997) has caused the research on ES to gradually become a hot topic in global ecological research [29]. In 2000, the launch of the MA once again promoted the development of ES research [30]. The project divided ES into four functions: Supply, culture, support and regulation [31]. It not only gave the scope and mode of ES value, but also pointed out the relationship between human well-being and ES [32]. The project defined ES as human benefits from natural ecosystems [33]. The evaluation methods of ES could be divided into two types: Value quantity and material quality evaluation [34].

At present, the use of remote sensing to assess ES has become a mainstream trend. It mainly carries out the key characteristic parameters of ecological assets, monitoring the changing trend at different times and the application of ES evaluation with the help of mature remote-sensing means and spatial analysis methods [35,36,37]. The ES assessment models that attracted scholars' attention include Multiscale Integrated Models of Ecosystem Services (MIMES) [38], the Global Unified Metamodel of the Biosphere (GUMBO) [39] and Artificial Intelligence for Ecosystem Services (ARIES) [40] developed by the University of Vermont, the Integrated Biosphere Simulator (IBIS) [41] developed by the University of Wisconsin, CITYgreen [42] developed by the U.S. Forestry Administration and the InVEST model developed by Stanford University [43]. Among them, the InVEST model has been more widely used because of its accurate quantification, visualization of results and low cost [44]. The InVEST model was mainly divided into marine, land and freshwater modules, and each module was specifically divided into several assessment projects. The assessment mainly included three aspects: The change in ES caused by the change in ecological environment dominated by the change in LUCC and landscape patterns [45]; crop yield evaluation, grassland resource evaluation and forest volume estimation aiming at evaluation and application [46]; and ES driven by ecosystem characteristic parameters, such as the net primary productivity, vegetation coverage and leaf area index [47].

The Mentougou District of Beijing is the western mountainous area of Beijing, the capital of China. As the western green barrier of the capital, it was also the only pure mountainous area in Beijing, providing important ES functions [48,49]. In the mountainous areas of the Chinese capital, land resources have economic, social, natural and ecological attributes [50,51]. This study took the Mentougou District of Beijing as the research object and took seven issues of remote-sensing images and other data (including terrain, landform, climate, soil, etc.) from 1985 to 2014 as the basic data source. Using the methods of supervised classification, landscape metrics, spatial statistical analysis, scenario simulation, grey-linear model, analytic hierarchy process and ES evaluation, the LUCC and ES function in the study area were used, evaluated and predicted. The purpose of this study was to (1) analyze the temporal and spatial transfer characteristics and landscape patterns of land use in the study area from 1985 to 2014; (2) carry out land suitability evaluation and scenario prediction and simulation of future land use; (3) evaluate the differences of ES functions in the study area in 2030 under different scenarios. The intention of the present study was to provide a scientific reference for the formulation of regional ecological environment construction and sustainable development.

## 2. Materials and Methods

### 2.1. Study Area

**Figure 1:**
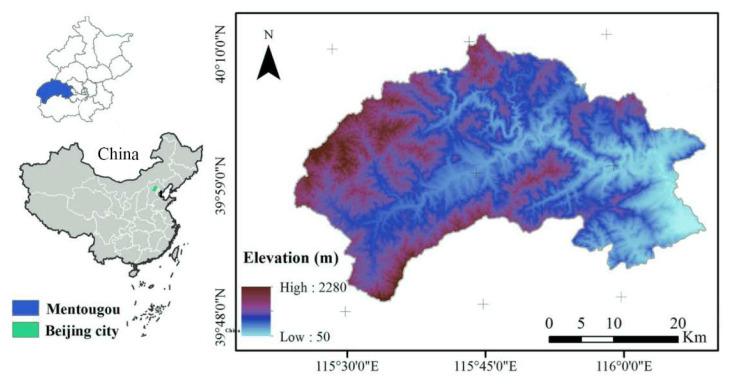
Location of Mentougou district and topography.

The Mentougou District is located in the northwest of Beijing, northern China (115°25′ E–116°10′ E, 39°48′ N–40°10′ N), with a total area of 1447.85 km^2^ (Figure 1). The region has a mid-latitude continental monsoon climate, and 98.5% of the region is mountainous [52]. In 2019, the average annual precipitation in Mentougou District was 405.7 mm and the average temperature was 13.8 °C [53]. Mentougou district has 4 streets and 9 towns, and in 2019, 254,000 people registered for residence and 344,000 permanent residents, of whom 42,000 belonged to the agricultural population and 211,000 to the non-agricultural population [54]. The regional GDP is 391.33 million dollars, and the primary, secondary and tertiary industries accounted for 1.24%, 26.94% and 71.84%, respectively [55].

### 2.2. LUCC Classification and Quantification of Landscape Patterns

In this study, we used remote-sensing image data of the Landsat Thematic Mapper (TM), Enhanced Thematic Mapper Plus (ETM) and Operational Land Imager (OLI) for nearly 30 years (one image per year from 1985, 1990, 1995, 2000, 2005, 2010 and 2014) [56,57]. Based on the Chinese land use classification system and the characteristics of the study area, land use is divided into nine types through supervised classification [58,59]. LUCC includes cropland (CL), orchard (OC), forestland (FL), shrubland (SL), grassland (GL), bareland (BL), waterbodies (WB), wasteland (WL) and built-up land (BUL), forming a grid image with a spatial resolution of 30 m (Table A1). Using the method of stratified random sampling, we randomly selected f 270 points (30 points for each land use type) in the study area, using higher-resolution remote-sensing images and related planning data (before 2005) and field survey (after 2005), and tested the accuracy of interpretation, and the overall accuracy of data interpretation for each year was over 85%.

#### 2.2.1. LUCC Transfer Matrix

**Figure 2:**
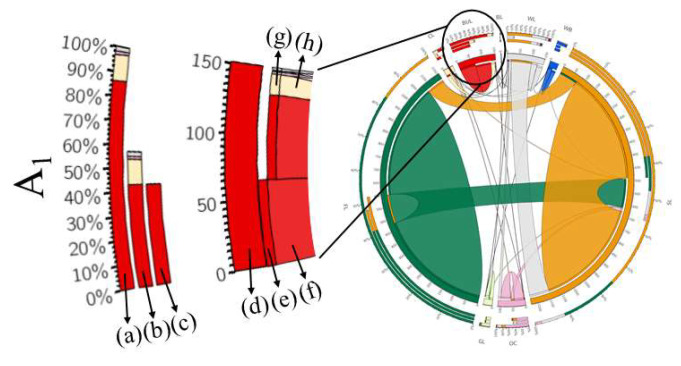
An explanation of the circos chart. Each stripe represents the occurrence of a transformation process at two different times, and the color represents the land use types. (**a**) The total amount of A_1_ transferred in and transferred out; (**b**) the proportion of each land-use type transferred into A_1_; (**c**) the proportion of each land-use type in transferred from A_1_; (**d**) the total area of A_1_ transformed out and transformed into other types; (**e**) the types of land use transformed from A_1_; (**f**) the area of A_1_ transformed into other land-use types; (**g**) the types of land use transformed into A_1_; (**h**) the area of A_1_ transformed from other land-use types.

Through the analysis of the land use transfer matrix, two different periods of transformation could be obtained. In order to clearly express the data of land use transfer from 1990 to 2015, we made a graph called circos and listed the data as an Appendix (Figure 2 and Table A2, Table A3, Table A4, Table A5, Table A6, Table A7 and Table A8). The mapping method using the template is from Canada’s Michael Smith Genome Sciences Centre (CMSGSC) (http://mkweb.bcgsc.ca/tableviewer/ accessed on 14 August 2021) [60,61].

#### 2.2.2. Statistical Analysis

To assess changes in the structural characteristics of landscape patterns at the sub-watershed level, we selected the number of patches (NP), mean patch size (MPS), patch density (PD) and patch cohesion index (COHESION), to characterize landscape patterns (Table 1) [62,63]. All calculations were extracted from the FRAGSTATS 4.2 software (Developed by the Clark Labs, Clark University).

### 2.3. AHP and CA-Markov

**Figure 3:**
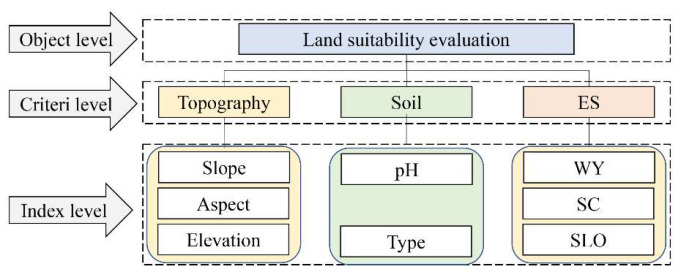
LUCC suitability evaluation structure map of Mentougou District.

According to ES demand and natural climate factors, a land suitability evaluation map was constructed (Figure 3). The analytic hierarchy process of determining the importance of each indicator was conducted by more than 20 experts in ecology-related fields. All results were tested for consistency using Yaahp software (developed by the Shanxi Yuan Decision Software Technology Co., LTD in China). Spatial simulation is based on the CA-Markov module in IDRIS 17.0 software (developed by the Clark Laboratory of Clark University in the United States) to simulate LUCC changes in the study area [64,65,66].

**Figure 4:**
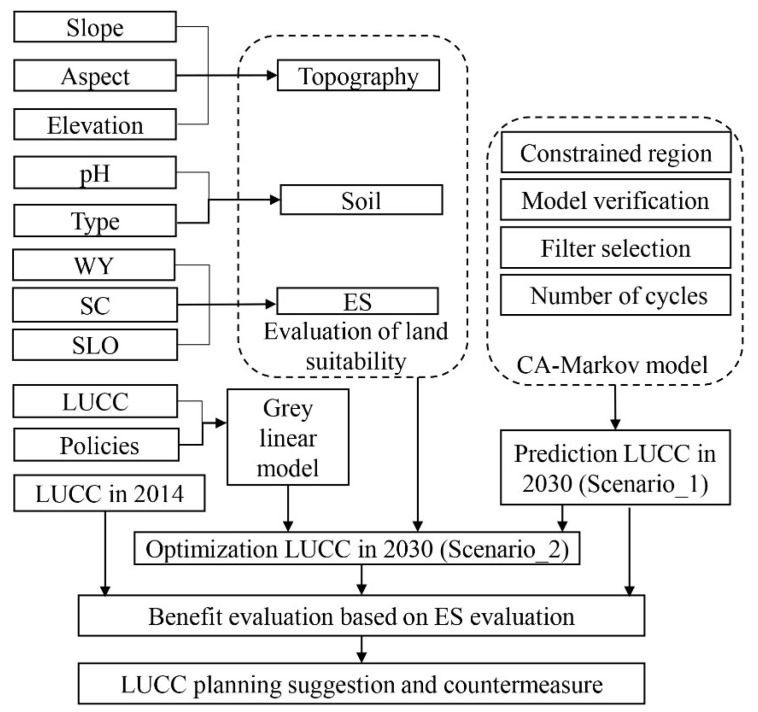
Optimization structure of LUCC.

The process was as follows (Figure 4): (1) Determine the transition area matrix and transition probability matrix of LUCC in the study area from 1985 to 2014, which were used to test the accuracy and predict the results with the CA-Markov model. (2) Then, we established the atlas of LUCC change suitability rules; the spatial distribution probability atlas of each LUCC was obtained by analyzing the limiting factors and driving factors as independent variables. (3) Next, the CA filter was constructed; in this study, a 5 × 5 mole neighborhood [21] (four adjacent cells above and below a cell are domains) was used as the filtering parameter of the CA-Markov model. (4) We then determined the starting time and the number of iterations. Firstly, the period from 1990 to 2010 was taken as the starting time of prediction, and the number of CA iterations was set as 10 to simulate the spatial distribution of land use types in the study area in 2010. Compared with the real results, kappa coefficients of all types of land use were > 0.70%, which was feasible. (5) Taking 1990 to 2014 as the starting point of prediction, the number of iterations was set as 16 to predict the LUCC in the study area in 2030. The prediction result was obtained (Scenario_1). (6) According to the land quantity structure and land suitability evaluation results, the land use transformation was restrained and controlled. The gray-linear model was used to solve the quantity of land use with equal emphasis on ecology and economy (Table A9). According to the results of the suitability analysis, priority should be given to the land use types requiring higher environmental conditions, and these types with higher land-use requirements should be allocated to the appropriate space, so as to obtain the optimization of the LUCC spatial distribution in 2030 (Scenario_2, regarding which land-use types are allocated to more suitable areas). (7) Lastly, the climate factors in 2014 were brought into the InVEST model to calculate and compare the ES of the predicted and optimized LUCC in 2030.

The kappa coefficient is calculated as follows [62]:

(1) $$\mathrm{Kappa}=\frac{P_{o}-P_{c}}{P_{p}-P_{c}}$$

where $P_{o}$ is the observed consistency rate between the reference data and the simulation data, $P_{c}$ is the expected correct simulation proportion in the case of randomness and $P_{p}$ is the correct simulation proportion in the case of ideal classification, which is generally 1.

### 2.4. Methods to Assessment ES

#### 2.4.1. Water Yield

The water yield (WY) module of the InVEST model can estimate the WY function of different LUCC. The core algorithm is used to calculate the WY of each grid by using the water balance method combined with climate, terrain and LUCC types. The WY is the precipitation of each grid in the region minus the actual evapotranspiration. The calculation formula of WY is as follows [67].

(2) $$Y_{x}=P_{x}-T_{AEx}$$

(3) $$T_{AEx}/P_{x}=\left(1+w_{x}R_{x}\right)/\left(1+w_{x}R_{x}+1/R_{x}\right)$$

(4) $$R_{x}=T_{PEx}/P_{x}$$

(5) $$C_{Kx}=T_{PEx}/T_{E0x}$$

(6) $$T_{AEx}=\min\left(C_{Kx}\times T_{E0x},P_{x}\right)$$

(7) $$w_{x}=Z\times \left(C_{Awx}/P_{x}\right)+1.25$$

$Y_{x}$ is the annual water yield of grid *x* (mm); $T_{AEx}$ is the actual annual evapotranspiration of grid *x* (mm); $P_{x}$ is the precipitation (mm) of grid *x* (mm); $T_{AEx}$ is approximated from the Budyko curve [67]; $R_{x}$ is the Budyko dryness index of grid *x*, which is the ratio of potential evapotranspiration ($T_{PEx}$) to precipitation ($P_{x}$); $C_{Kx}$ is the evapotranspiration coefficient of vegetation, which is different in different vegetation types, and represents the ratio of potential evapotranspiration to reference evapotranspiration ($T_{E0x}$) of plants at different growth stages; $T_{AEx}$ is evaluated directly by $T_{E0x}$, and its value is determined jointly by $T_{E0x}$ and $P_{x}$; $C_{Awx}$ is the ratio of soil water availability to precipitation; $C_{Awx}$ is the soil available water content of grid *x* (mm); *Z* is the seasonal constant.

#### 2.4.2. Soil Conservation and Soil Loss

The Sediment Delivery Ratio model of InVEST is used to calculate soil loss and soil conservation based on the soil loss equation. The original soil loss equation is added to the model to intercept the sediment and grid upstream sediment, and on this basis, the soil conservation amount is calculated [68].

(8) $$RUSLE_{n}=R_{n}\times K_{n}\times LS_{n}\times C_{n}\times P_{n}$$

(9) $$USLE_{n}=R_{n}\times K_{n}\times LS_{n}$$

(10) $$SEDRET_{n}=USLE_{n}-RUSLE_{n}+SED_{n}$$

where $RUSLE_{n}$ is the actual amount of soil erosion, $USLE_{n}$ is the potential soil loss under no vegetation coverage, $SEDRET_{n}$ is the soil conservation, $R_{n}$ is the rainfall erosivity, $K_{n}$ is the soil erodibility factor, $LS_{n}$ is the slope length–gradient factor, $C_{n}$ is the crop management factor, $P_{n}$ is the support practice factor, and $SED_{n}$ is the amount of sediment and sediment intercepted upstream.

#### 2.4.3. Carbon Stocks

The Carbon stocks model of InVEST quantifies carbon storage and sequestration based on four carbon pools: Aboveground biomass, underground biomass, dead organic matter and soil organic matter [69,70]. Carbon storage in vegetation is estimated by multiplying the evegetation carbon density by the area of each LUCC based on local research results [71].

The carbon density was cropland (30.96 tC·ha^−1^), orchard (30.96 tC·ha^−1^), forested land (150.40 tC·ha^−1^), scrubland (118.42 tC·ha^−1^), grassland (96.68 tC·ha^−1^), bare land (10.00 tC·ha^−1^), water bodies (0 tC·ha^−1^), wasteland (96.68 tC·ha^−1^) and built-up land (0 tC·ha^−1^), respectively, according to the related reference [49].

(11) $$C_{total}=C_{above}+C_{below}+C_{soil}+C_{dead}$$

where $C_{total}$ is the total carbon stocks (t/hm^2^), $C_{above}$ is the aboveground carbon stocks t/hm^2^), $C_{below}$ is the underground carbon stocks (t/hm^2^), $C_{soil}$ is the soil carbon stocks (t/hm^2^), and $C_{dead}$ is the dead organic carbon stocks (t/hm^2^).

## 3. Results

### 3.1. Transfer Characteristics of LUCC and Landscape Patterns

#### 3.1.1. Temporal and Spatial Transfer Characteristics of LUCC

**Figure 5:**
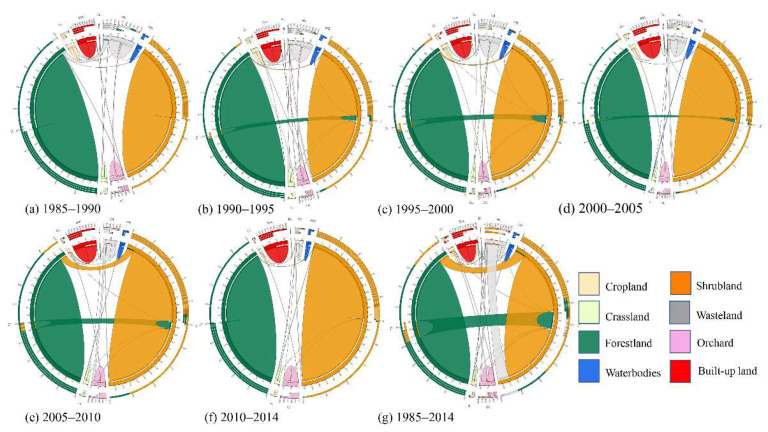
LUCC transfer flow charts in the Mentougou District from 1985 to 2014.

During the study period, the total BUL increased by 21.11 km^2^ (32.40%). This increased BUL was mainly the cost of CL (15.49 km^2^), OC (2.58 km^2^) and WL (2.42 km^2^). During the study period, the CL decreased rapidly, with a total decrease of 66.97% (20.13 km^2^). Before 2000, the reduced area of CL was mainly transformed into BUL and WL. After 2000, CL was mainly transformed into OC and BUL. Before 2000, the transferred CL area was 18.80 km^2^, of which 53.14% (9.99 km^2^) was converted to BUL and 43.24% (8.15 km^2^) was converted to WL. From 2000 to 2010, the total area of CL transferred out was 14.94 km^2^. Of the reduced CL, 32.20% (4.81 km^2^) was transformed into BUL and 42.24% (6.31 km^2^) into OC. In general, in the past 30 years, the urbanization development of the study area was mainly manifested in the transformation of agricultural land (CL and OC) into BUL. CL accounts for 73.38% of the total land transformed into BUL and OC accounts for 12.22%. In addition, the WL also decreased significantly by 80.87%, mainly transformed into BUL, FL, SL and OC, of which the area converted to FL accounted for 2.58%, BUL accounted for 2.36%, OC accounted for 10.85% and SL accounted for 82.03%. The transfer into SL comprised 224.94 km^2^ and the transfer out SL was 74.82 km^2^. The area transferred into FL comprised 77.43 km^2^ and the area of FL transferred out was 120.46 km^2^ (Figure 5).

#### 3.1.2. Evolution Characteristics of Landscape Patterns of LUCC

**Figure 6:**
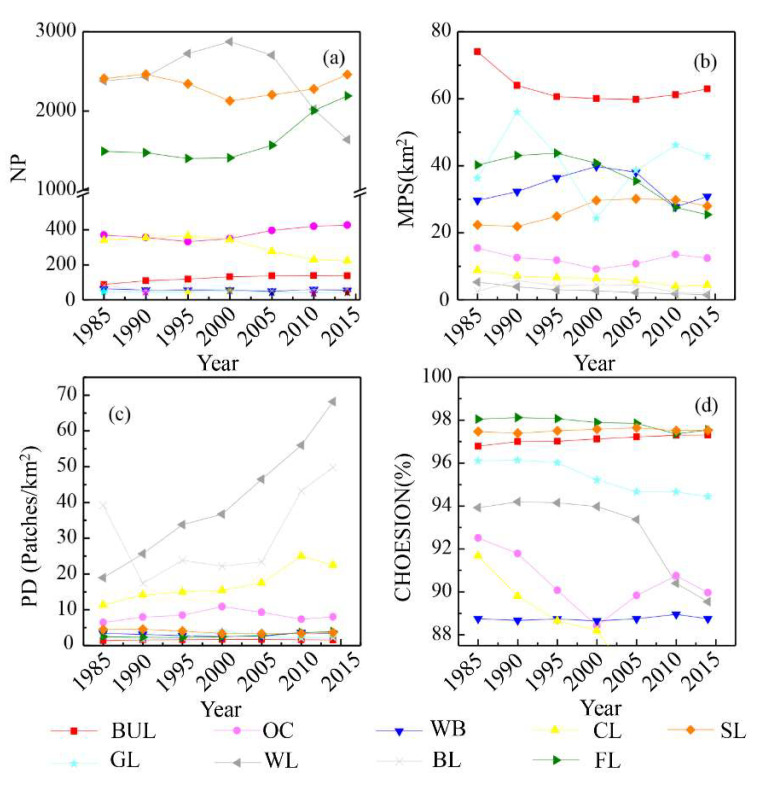
Landscape pattern changes of LUCC in Mentougou District. (**a**) NP; (**b**) MPS; (**c**) PD; (**d**) CHOESION. The types of LUCC were built-up land (BUL), orchard (OC), waterbodies (WB), cropland (CL), shrubland (SL), grassland (GL), wasteland (WL), bare land (BL) and forestland (FL).

During the study period, BUL became more fragmented, and landscape heterogeneity increased. The landscape heterogeneity of OC first increased and then decreased. Before 2000, the landscape patterns of OC showed a trend of fragmentation, and after 2000, landscape connectivity increased. CL and FL became fragmented, reducing the connectivity. There were no obvious changes in WB, GL and BL during the study period. The PD of BUL increased from 1.35 patches/km^2^ to 1.59 patches/km^2^. The NP and MPS of CL decreased from 1985 to 2014, reaching 340 patches (8.84 × 10^−2^ km^2^) and 224 patches (4.43 × 10^−2^ km^2^), respectively (Figure 3). The NP of WL increased first and then decreased, from 2379 in 1985 to 2873 in 2000, and then decreased to 1639 in 2014. The MPS and PD of WL decreased, and the COHESION decreased. The NP of SL increased from 1491 patches in 1985 to 2189 patches in 2014. The NP of SL decreased first and then increased, from 2409 patches in 1985 to 2128 patches in 2000, and then increased to 2460 patches in 2014 (Figure 6).

### 3.2. Spatial Distribution of ES and Eco-Environmental Suitability of LUCC

#### 3.2.1. ES of Mentougou District in 2014

**Figure 7:**
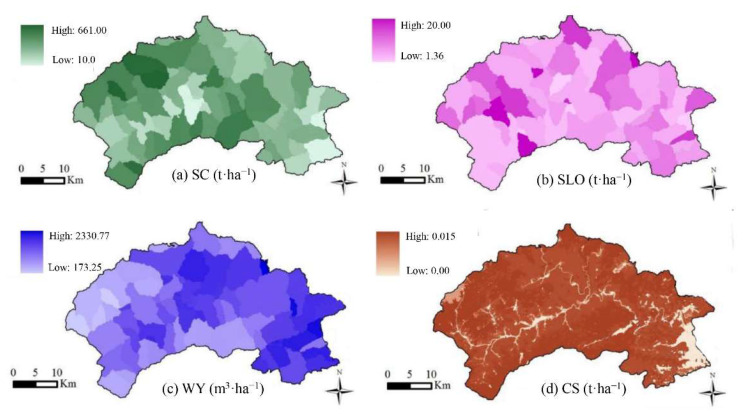
Spatial distribution of soil conservation (SC) (**a**), soil loss (SLO) (**b**), water yield (WY) (**c**) and (**d**) carbon stocks (CS) by InVEST in 2014 of Mentougou District.

The total amounts of SC, SLO, WY and CS in 2014 were 0.50 × 10^8^ t, 0.66 × 10^6^ t, 2.02 × 10^8^ m^3^ and 1713.54 × 10^4^ t, respectively. In terms of spatial characteristics, CS, SC and SLO were mainly distributed in some hilly areas in Mentougou District, followed by hilly areas with relatively fragmented LUCC along the river. This part of LUCC was mainly composed of CL, OC and WL. The smallest area was mainly concentrated in the southeast Mentougou District plain area and part of the BL. In general, the CS and WY of different LUCC vary greatly. FL and SL had the strongest ES capacity, with a total CS of 1.73 × 10^8^ m^3^ (85%), SC of 0.43 × 10^8^ t (80%), SLO of 0.46 × 10^6^ t (70%), and WY of 0.16 × 10^8^ t (96.48%) (Figure 7).

#### 3.2.2. Spatial Distribution and Quantitative Structure of Eco-Environment

**Figure 8:**
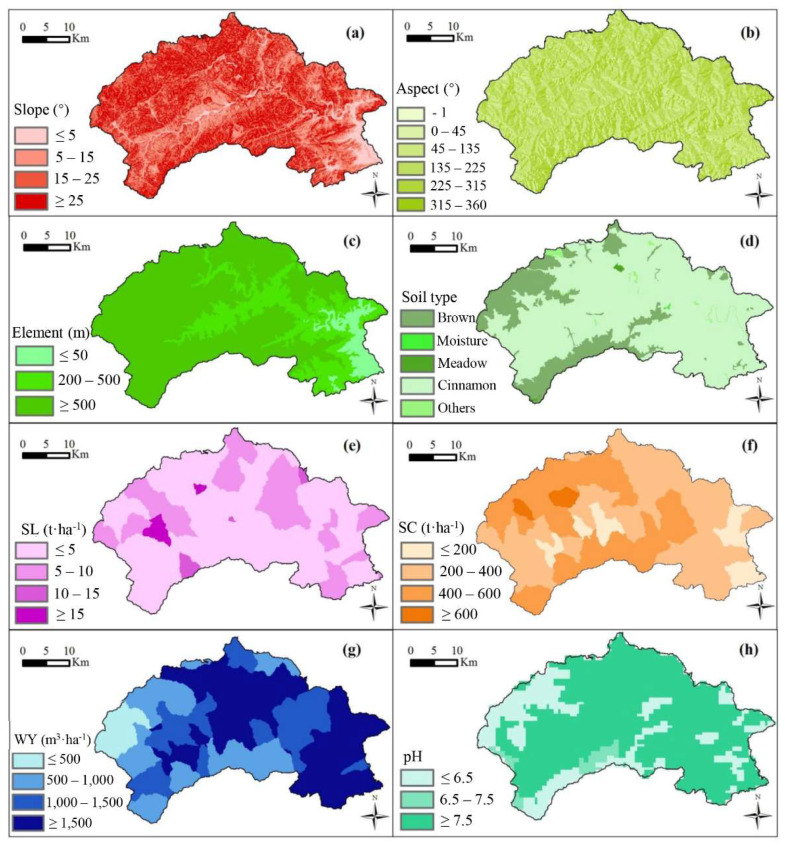
Map of evaluating indicator distribution in Mentougou District; (**a**–**d**) the slope, aspect, elevation and soil type; (**e**–**h**) the soil loss, soil conservation, water yield and soil pH.

According to geomorphology, the study area is mainly divided into three types: Plain area (≤200 m), hilly area (200–500 m) and mountainous area (≥500 m), covering 85.00 km^2^ (5.84%), 333.15 km^2^ (22.90%) and 85.00 km^2^ (71.26%), respectively. The main soil types were cinnamon and brown, accounting for 1136.32 km^2^ and 298.93 km^2^ or 78.10% and 20.55%, respectively. The area of bare land and water area was 14.48 km^2^, covering an area of 1.00% (Figure 8).

Mentougou district has a total area of 107.59 km^2^ with a slope between 0° and 5°, accounting for 7.40% of the whole region. An area of 386.62 km^2^ has a slope between 5° and 15°. An area of 475.07 km^2^ has a slope between 15° and 25°, accounting for 32.65%. The area with a slope of >= 25° was 485.60 km^2^, accounting for 33.38% of the county area. The sunny slope accounts for 25.01% of the total area of the district, and the shady slope for 38.62%. Soil pH was nearly neutral with values between 6.5 and 7.5, covering a total of 1027.39 km^2^, accounting for 70.62% of the whole region. The acidic soil (pH 5.5~6.5) covers an area of 395.5 km^2^, covering 27.18%. The area of alkaline soil (pH 7.5~8.5) was 31.99 km^2^, covering 2.20%.

**Figure A1:**
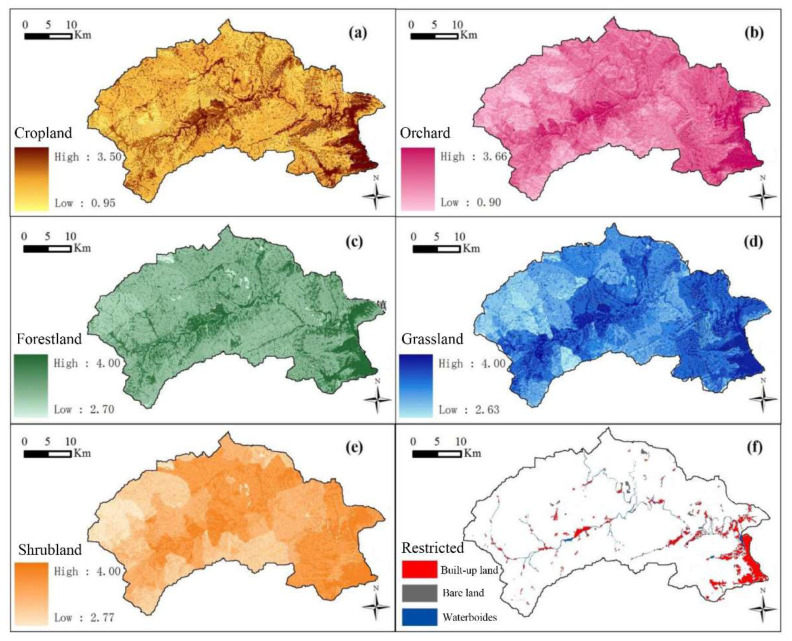
Map of land use type suitability evaluation in Mentougou District; (**a**–**e**) the spatial distribution of suitability for cropland, orchard, forestland, grassland and shrubland; (**f**) areas that are not included in model prediction.

The area with SLO less than or equal to 10 t/hm^2^ had a total of 1405.77 km^2^ (96.63%). The area with SC less than or equal to 200 t/hm^2^ was 140.06 km^2^, accounting for 9.63% of the total area of the whole region. The area of SC between 200 t/hm^2^ and 600 t/hm^2^ was 1281.19 km^2^, covering 88.06%. The area with WY less than or equal to 500 m^3^/hm^2^, 500~1000 m^3^/hm^2^, 1000~1500 m^3^/hm^2^ and greater than or equal to 1500 m^3^/hm^2^ were 84.63 km^2^ (5.82%), 361.03 km^2^ (24.82%), 375.98 km^2^ (25.84%) and 633.24 km^2^ (43.53%), respectively. The evaluation was rated on a scale of 0 to 2, 2 to 3 and 3 to 4, which were suitable, relatively suitable and unsuitable, respectively (Table A7 and Figure A1).

#### 3.2.3. Spatial Distribution Characteristics of Suitable LUCC

**Figure 9:**
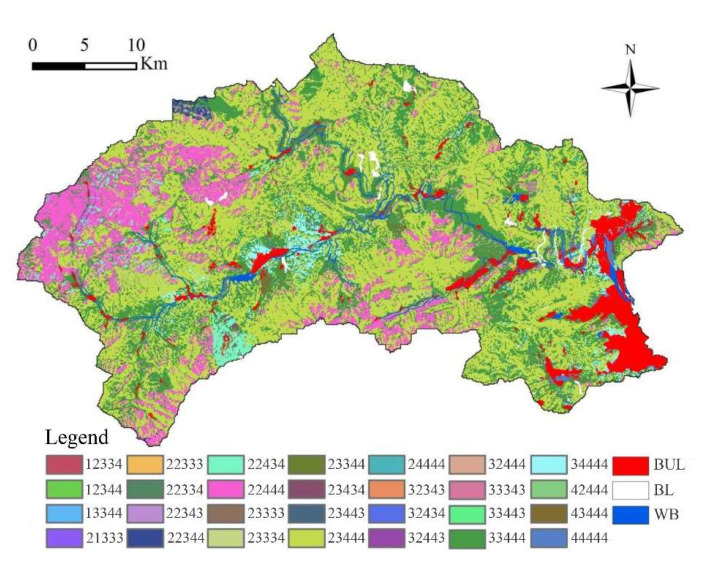
Land suitability evaluation map in Mentougou District. Note: The five digits of each legend, from left to right, represent cropland, orchard, forestland, grassland and shrubland. The score of each digit is 4, 3, 2 and 1; 4 represents the suitable distribution area of a certain land use type; 3 represents the relatively suitable distribution area of a certain land use type; 2 and 1 represent the unsuitable distribution area of a certain land-use type.

The results of the land suitability grade evaluation showed that the area suitable for CL was 514.36 km^2^ (35.35%), mainly distributed in the gully and hilly regions (Figure A1 and Table A10). The area unsuitable for CL was 940.52 km^2^, accounting for 64.65% of the total area of the whole region (Figure 9 and Table 2). The area suitable for OC was 1130.64 km^2^ (77.71%), mainly distributed in the eastern plain and part of the central hilly region, mainly in most plain and hilly areas, and some mountainous areas. The area of suitable FL and relatively suitable FL was 1350.94 km^2^ (92.86%). The area of suitable GL and relatively suitable GL was 1350.94 km^2^ (92.85%). The area unsuitable for SL was 103.94 km^2^ (7.14%).

### 3.3. Comparative Results of LUCC and ES under the two Scenarios

#### 3.3.1. LUCC Prediction and Optimization Results in 2030

**Figure 10:**
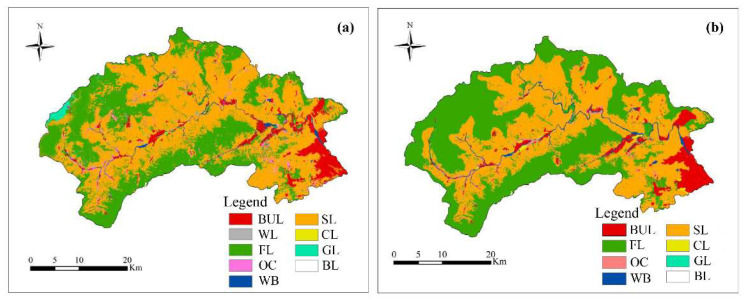
LUCC of prediction (Scenario_1: Prediction of 2030) (**a**) and optimization (Scenario_2: Optimization of 2030) (**b**) of Mentougou in 2030.

The comparison between Scenario_1 in 2030 and the situation in 2014 showed that BUL will mainly expand into adjacent areas in the future. The difference was that the patch size and expansion rate of each BUL were different. The spread degree of BUL in plain areas was higher than that in the hilly areas, and the spread degree of BUL was the slowest in mountainous areas (Figure 10). Compared with the LUCC situation in 2014, all the WL in the Scenario_2 (Optimization of 2030) was changed into other LUCC with higher comprehensive benefits, and the WL in the prediction results increased by 1.22 km^2^ (5.07%). The area of FL and WB increased by 22.91 km^2^ (4.12%) and 5.88 km^2^ (35.74%), respectively, while the area of CL and SL had little change. The FL, OC and WB decreased by 53.47 km^2^ (9.61%), 11.78 km^2^ (22.14%) and 0.28 km^2^ (1.70%), respectively, and the BUL increased in both scenarios. In terms of spatial distribution, Cl and OC migrate from an unsuitable area to a suitable area (Figure 10 and Table 3).

#### 3.3.2. Comparison of the ES of Present, Prediction and Optimization

The predicted and optimized land use map of 2030 was used to predict the model, together with the meteorological data and soil data of 2014 (precipitation, potential evapotranspiration, water available to vegetation, soil depth and rainfall erosivity, etc.). The predicted and optimized land use ES was calculated and compared with the ES in 2014.

**Figure 11:**
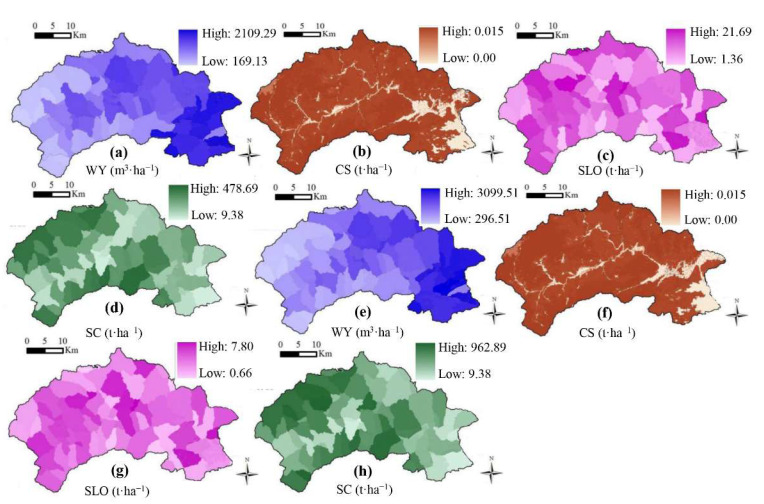
Spatial distribution of ES after prediction and optimization by CA-Markov and InVEST; (**a**–**d**) the ES as predicted for 2030; (**e**–**h**) the ES as optimization for 2030.

From the modelled data, in 2030. all ecosystem functions were declining. WY, CS and SC decreased by 0.16 × 10^8^ m^3^ (7.92%), 6.15 × 10^4^ t (0.36%) and 0.08 × 10^8^ t (16.00%), while SLO increased by 0.08 × 10^6^ t (12.12%) (Figure 11 and Table 4). After land-use optimization, all ES except CS would be improved. WY would be increased by 31.19% (0.63 × 10^8^ m^3^) and SC would be increased by 12.00% (0.06 × 10^8^ t). SLO would be decreased by 9.09% (0.06 × 10^6^ t). However, CS would be decreased slightly, by 0.35% (Figure 11 and Table 4). In general, at the optimized land use, WY and SC capacity would be improved, and ES would be improved (Figure 11 and Table 4).

## 4. Discussion

### 4.1. Impacts of Human Activities and Policies on LUCC

The landscape patterns of Mentougou District have undergone great changes. Since the promulgation of the Land Management Law in 1986, the market economy system (an economy in which social resources were allocated through the market) have gradually occupied a dominant position [72]. Mentougou District lost more than half of its CL in the nearly 30 years from 1985 to 2014. People tend to choose more fertile land for farming and thus the productivity of the land decreased distinctly during this period [73,74,75]. Meanwhile, BUL increased steadily during the study period. Such changes are common in rapidly urbanizing suburbs, and the Los Angeles and California suburbs of Orange County and San Bernardino County have witnessed a dramatic loss of prime farmland and orange groves to suburban development [76]. The study area implemented the policy “Grain to Green” in 1999, prompting further losses of cropland [49]. Future economic development should take into account the unique natural conditions of mountainous areas and their landscape patterns. The government should adopt development and land management policies so that new urban development can be sustainable.

### 4.2. The Impact of Landscape Patterns Changes

The WL in Mentougou District was gradually fragmented, especially after 2005, and landscape heterogeneity increased rapidly. The BUL, CL and BL showed a trend of fragmentation in this period, and the degree of landscape aggregation decreased. The plain sub-region of Mentougou District accounted for less than 10% of the region’s area but contained more than 60% of the population [50]. People preferred to build houses in the plains, near the existing residential development [17]. Population pressure in the plains decreased the area of cropland as it was converted to built-up land. Over time, small patches of FL increased, and SL formed large patches, which became the main LUCC in the study area. The landscape heterogeneity of OC and WB increased first and then decreased. Before 2000, the landscape patterns of OC were fragmented, and after 2000, landscape connectivity increased. The habitat was divided into fragmentary patches, which reduced ecosystem stability and accelerated the invasion of alien species due to the edge effect. Since 2005, invasive alien species have been found in Mentougou District, including *Solanum rostratum* Dunal. and *Pueraria phaseoloides* (Roxb.) Benth. [77]. These alien species, with their strong ability to survive, will take over the resources of other plants and animals and cause the extinction of local species [78,79].

### 4.3. Spatial Suitability of Various Types of LUCC in the Region

Through the classification and evaluation of Mentougou’s basic eco-environmental factors (including slope, aspect, elevation, soil type, soil pH and ES), the suitable spatial distribution positions and quantities of various LUCC in the study area were selected. According to the Chinese policy of Grain to Green, areas with a slope greater than 25° were strictly not allowed to be CL, so the suitable area for CL accounted for only one-third of the total area [80,81]. The suitable area of CL was mainly distributed in the eastern plain area and some parts of the central hilly area. These places were usually rich in water and experienced less soil loss [82]. The CL-suitable area was mostly brown soil and tidal soil, with a small slope, complete water conservancy facilities, convenient irrigation and high management level. It was a basic farmland area with high and stable output in Mentougou District. The unsuitable area of OC accounted for 22.29%. Cinnamon accounts for more than two-thirds of the study area, and most areas were suitable for FL, GL and SL. After returning CL to FL, the FL in the study area would further increase. The FL could improve the regional microclimate, reduce soil erosion and water evaporation, prevent wind and consolidate soil and improve soil physical and chemical properties. However, our study did not compare different suitability evaluation methods. Future studies should add other evaluation methods, such as machine learning and sensitivity analysis, to increase the objectivity of suitability evaluation [83,84].

### 4.4. Impacts of Future LUCC on ES

Based on the existing spatial transfer matrix of LUCC, the CA-Markov model was used to predict LUCC in 2030, the control constraints were set and the Scenario_2 spatial distribution map of LUCC in 2030 was obtained. Compared with the LUCC in 2014, Scenario_2 was more in line with the protection needs of the local government for CL. From the goal of common development of ecological benefits (ecological space) and economic benefits (built-up land space), Science_2 is more suitable than Science_1. Linking the InVEST and CA-Markov models has promising applications for guiding ecological engineering. First, the CA-Markov model was applied to simulate LUCC by combing eco-environmetat, which provides a method for simulating LUCC in other areas [17,26]. More importantly, the predicted land cover map aided in the identification of areas where ecological space is more likely to occur, which has great significance for site selection for investment in ecological engineering. Second, the InVEST model can quantify a number of ecosystem services [44,46].

Against the background of the south-to-north water transfer and the government's encouragement of ecological restoration of mountains, rivers, forests, farmland and lakes, Scenario 2 would provide important guidance for planning. In addition, the OC of Scenario_1 was reduced by 11.78 km^2^ (22.14%) and the OC of Scenario_2 remained unchanged. In Scenario_1, SL increased, while in Scenario_2, SL remained unchanged. Because the economic and ecological benefits of the GL in the study area were not high, the area was reduced in both Scenario_1 and Scenario_2 (Table 3). In terms of spatial distribution, Scenario_2 was more conducive to the future development of the research area. ES (WY, CS, SC and SL) was evaluated using environmental and climatic factors in 2014, and each index proved that Scenario_2 had higher ES capacity.

## 5. Conclusions

From the perspective of landscape patterns, during the past 30 years (1985–2014), LUCC in Mentougou District had undergone drastic changes, with rapid urbanization. BUL increased by one-third, CL decreased by more than half, and WL decreased by more than two-thirds. At the same time, the landscape patterns of various LUCC also changed, generally showing a trend of gradual fragmentation, decreasing landscape connectivity and increasing heterogeneity. In terms of ES and spatial suitability, the total CS, SC, SLO and WY in the study area in 2014 were 1713.54 × 10^4^ t, 0.50 × 10^8^ t, 0.66 × 10^6^ t and 2.02 × 10^8^ m^3^. The area suitable for CL and OC in the study area accounted for one-third and two-thirds of the whole study area, and the area suitable for FL, GL and SL accounted for more than 90%. In 2030, the areas of FL, WB, OC and CL in the study area would decrease in Scenario_1, and CL would especially decrease by about half. In Scenario_2, FL and WB would increase by about 40% in total, and BUL also would develop well. In scenario_2, ES would be also better than scenario_1, WY and SC would increase by more than one-third and SLO would decrease. According to the land-use configuration of Scenario_2, the vegetation would grow under more suitable environmental conditions, and the spatial distribution of land use would be more reasonable. This study is helpful for policymakers, planners and landscape designers to determine urban land use schemes and promote the rational allocation of urban land.

## Figures and Tables

**Table 1:** Description of landscape metrics.

Types	Model Name	Calculation Method	Connotation
Area/ Density	NP	$NP=n_{i}$	The number of patches in landscape.
	PD	$PD=\frac{n_{i}}{a_{ij}}\left(10000)(100\right)$	The density of landscape
	MPS	$MPS=\overset{n}{\underset{j=1}{\sum }}\frac{a_{ij}}{n_{i}}$$(\frac{1}{10000}$)	It can be used to characterize landscape fragmentation.
Aggregation	COHESION	$COHESION=\frac{1-\frac{\sum_{j=k}^{n}p_{ik}}{\sum_{j=1}^{n}p_{ij}\sqrt[{n}]{a_{ij}}}}{1-\frac{1}{\sqrt{n_{i}}}}\left(100\%\right)$	It reflects the aggregation degree of patches in the landscape.

NOTE: $a_{ij}$ is the area of patch ij, $p_{ij}$ is the common border length of patch *ij*, and $n_{i}$ is the number of patches in the landscape of patch type (lass) i.

**Table 2:** The main LUCC suitability structure in Mentougou District (km^2^).

Type	CL	OC	FL	GL	SL
Appropriate	16.45	27.79	1341.78	1331.26	1346.02
Relatively appropriate	497.91	1102.86	9.16	19.68	4.92
Inappropriate	940.52	324.23	103.94	103.94	103.94

**Table 3:** Area of LUCC in present situation, prediction and optimization in Mentougou (km^2^).

LUCC	2014	Prediction of 2030 (Scenario_1)	Optimization of 2030 (Scenario_2)
CL	9.93	5.07	9.93
OC	53.21	41.43	53.21
FL	556.54	503.07	579.45
SL	689.23	759.39	689.23
GL	17.98	13.05	0.19
WB	16.45	16.17	22.33
BUL	86.27	90.81	99.32
WL	24.05	25.27	0.00
BL	1.22	0.61	1.22

**Table 4:** The ES of present situation, prediction and optimization.

ES	2014	Prediction for 2030 (Scenario_1)	Optimization for 2030 (Scenario_2)
WY(m^3^)	2.02 × 10^8^	1.86 × 10^8^	2.65 × 10^8^
CS(t)	1713.54 × 10^4^	1707.39 × 10^4^	1707.53 × 10^4^
SC(t)	0.50 × 10^8^	0.42 × 10^8^	0.56 × 10^8^
SLO(t)	0.66 × 10^6^	0.74 × 10^6^	0.60 × 10^6^

## Appendix A

**Table A1:** Abbreviations of land use and land coverage changes (LUCC), landscape metrics (LM) and ecosystem service (ES).

Types	Abbreviation	Content
Land use and land coverage changes (LUCC)	BUL	Built-up land
	OC	Orchard
	WB	Water bodies
	CL	Cropland
	GL	Grassland
	WL	Wasteland
	BL	Bareland
	FL	Forestland
	SL	Shrubland
Landscape metrics (LM)	NP	Number patches
	PD	Patch density
	MPS	Mean Patch Area
	COHESION	Patch Cohesion Index
Ecosystem service (ES)	WY	Water yield
	SLO	Soil Loss
	SC	Soil conservation
	CS	Carbon stocks

**Table A2:** Land use transfer matrix in Mentougou District from 1985 to 1990 (km^2^).

	BUL	OC	WB	CL	GL	WL	BL	FL	SL
BUL	65.16								
OC	1.37	44.99				8.41		2.4	
WB			18.2			0.45			
CL	3.07			23.01		3.98			
GL					17.07				
WL	0.43			2.06	7.18	82.02	1.93	32.09	
BL					0.98		1.11		
FL								599.58	
SL								0.8	538.59

NOTE: The data in each row add up to the total area of land use in the same category in 1985, and the data in each column equal the total area of land use in the same category in 1990. The data in each cell represent the area of the row land-use type transferred to the column land-use type from 1985 to 1990. The total area of the study area is 64,500 km^2^.

**Table A3:** Land use transfer matrix in Mentougou District from 1990 to 1995 (km^2^).

	BUL	OC	WB	CL	GL	WL	BL	FL	SL
BUL	70.03								
OC	0.83	34.13		0.87		7.86			1.3
WB			18.2						
CL	0.4			20.52		4.15			
GL					18.01			0.28	6.94
WL	0.14	5.29		2.46	0.62	68.6		8.36	9.39
BL					0.45		2.59		
FL				0.54				600.7	33.63
SL			1.84					3.76	532.99

NOTE: The data in each row add up to the total area of land use in the same category in 1990, and the data in each column equal the total area of land use in the same category in 1995. The data in each cell represent the area of the row land-use type transferred to column land-use type from 1990 to 1995. The total area of the study area is 64,500 km^2^.

**Table A4:** Land use transfer matrix in Mentougou District from 1995 to 2000 (km^2^).

	BUL	OC	WB	CL	GL	WL	BL	FL	SL
BUL	71.39								
OC		32.01				2.75			4.66
WB			20.04						
CL	6.52	0.17		17.17					0.51
GL					12.59	6.49			
WL	1.07			5.14		68.95		1.08	4.38
BL					0.06		2.53		
FL								563.65	49.46
SL			2.29			0.01		9.92	572.04

NOTE: The data in each row add up to the total area of land use in the same category in 1995, and the data in each column equal the total area of land use in the same category in 2000. The data in each cell represent the area of the row land-use type transferred to column land-use type from 1995 to 2000. The total area of the study area is 64,500 km^2^.

**Table A5:** Land use transfer matrix in Mentougou District from 2000 to 2005.

	BUL	OC	WB	CL	GL	WL	BL	FL	SL
BUL	78.98								
OC		32.18							
WB			18.76		3.57				
CL	2.49	4.02		15.8					
GL					12.65				
WL	0.48	6.31			0.39	58.13			12.89
BL							2.21		0.32
FL								554.01	20.63
SL								0.91	630.15

NOTE: The data in each row add up to the total area of land use in the same category in 2000, and the data in each column equal the total area of land use in the same category in 2005. The data in each cell represent the area of the row land-use type transferred to column land-use type from 2000 to 2005. The total area of the study area is 64,500 km^2^.

**Table A6:** Land use transfer matrix in Mentougou District from 2005 to 2010.

	BUL	OC	WB	CL	GL	WL	BL	FL	SL
BUL	81.96								
OC		38.03							4.48
WB			16.34	1.05	1.37				
CL	2.32	2.29		7.37					3.82
GL					15.78				0.82
WL	0.39	16.77		0.77		36.11			4.09
BL					0.85		1.36		
FL								502.31	52.61
SL								51.01	612.98

NOTE: The data in each row add up to the total area of land use in the same category in 2005, and the data in each column equal the total area of land use in the same category in 2010. The data in each cell represent the area of the row land-use type transferred to column land-use type from 2005 to 2010. The total area of the study area is 64,500 km^2^.

**Table A7:** Land use transfer matrix in Mentougou District from 2010 to 2015.

	BUL	OC	WB	CL	GL	WL	BL	FL	SL
BUL	84.67								
OC		53.19		0.74					3.16
WB			16.17			0.17			
CL	1.6			7.59					
GL					16.89				1.11
WL		0.02	0.28	1.6	0.66	23.88			9.67
BL					0.14		1.22		
FL								553.32	
SL					0.29			3.22	675.29

NOTE: The data in each row add up to the total area of land use in the same category in 2010, and the data in each column equal the total area of land use in the same category in 2015. The data in each cell represent the area of the row land-use type transferred to column land-use type from 2010 to 2015. The total area of the study area is 64,500 km^2^.

**Table A8:** Land use transfer matrix in Mentougou District from 1985 to 2015.

	BUL	OC	WB	CL	GL	WL	BL	FL	SL
BUL	65.16								
OC	2.58	39.59		1.77					13.23
WB	0.62		16.42	0.05	0.94	0.62			
CL	15.49	2.51		7.98		0.12			3.96
GL					13.79				3.28
WL	2.42	11.11		0.13	1.73	23.3	0.37	2.64	84.01
BL					1.24		0.85		
FL								479.12	120.46
SL			0.03					74.79	464.57

NOTE: The data in each row add up to the total area of land use in the same category in 1985, and the data in each column equal the total area of land use in the same category in 2015. The data in each cell represent the area of the row land-use type transferred to column land-use type from 1985 to 2015. The total area of the study area is 64,500 km^2^.

**Table A9:** The LUCC quantitative structure obtained using grey-linear model.

Scenario_2	CL	OC	FL	SHL	GL	WB	BUL	WL	BL
Quantitative of LUCC	9.93	53.21	579.45	689.23	0.19	22.33	99.32	0.00	1.22

**Table A10:** The structure table of evaluating indicators in Mentougou District.

Factors	Types	Area (km^2^)	Percentage (%)	Suitability Evaluation						
				Cropland	Orchard	Forestland	Grassland	Shrubland	Legend	
Slope(°)	≤5	107.59	7.4	4	4	4	4	4		High
	5~15	386.62	26.57	3	3	4	4	4		
	15~25	475.07	32.65	0	2	3	3	4		
	≥25	485.6	33.38	0	0	2	0	2		
Aspect(°)	0°	6.48	0.45	4	4	4	4	4		Low
	0°–45°	194.21	13.35	2	1	4	3	4		
	45°–135°	392.64	26.99	3	3	3	4	3		
	135°–225°	363.82	25.01	4	4	4	4	4		
	225°–315°	324.13	22.28	3	3	3	4	3		
	315°–360°	173.61	11.93	2	1	4	3	4		
Elevation(m)	≤200	85	5.84	4	4	4	4	4		
	200~500	333.15	22.9	3	3	4	4	4		
	≥500	1036.73	71.26	1	2	4	4	4		
Soil type	Soilless area	14.48	1	0	0	0	0	0		
	Cinnamon soil	1136.32	78.1	3	3	4	4	4		
	Brown soil	298.93	20.55	4	4	4	4	4		
	Moisture soil	0.88	0.06	4	4	4	4	4		
	Meadow soil	4.27	0.29	0	3	2	4	2		
Soil Conservation(t/hm^2^)	≤200	140.06	9.63	3	3	4	4	4		
	200~400	738.19	50.74	2	2	4	4	4		
	400~600	543	37.32	1	1	4	3	4		
	≥600	33.64	2.31	0	0	4	2	4		
Water Yield(m^3^/hm^2^)	≤500	84.63	5.82	0	0	0	2	1		
	500~1000	361.03	24.82	1	2	2	3	2		
	1000~1500	375.98	25.84	4	3	3	3	3		
	≥1500	633.24	43.53	4	4	4	4	4		
Soil loss(t/hm^2^)	≤5	981.68	67.48	4	4	4	4	4		
	5~10	424.09	29.15	3	3	4	3	4		
	10~15	22.91	1.57	1	2	4	2	4		
	≥15	26.2	1.8	0	0	4	1	4		
pH of soil	5.0~6.5	395.5	27.18	0	0	3	3	3		
	6.5~7.5	1027.39	70.62	4	4	4	4	4		
	7.5~8.5	31.99	2.2	2	2	3	3	3		
